# Populations of large-diameter trees are increasing across the United States

**DOI:** 10.1073/pnas.2421780122

**Published:** 2025-03-10

**Authors:** Paul J. Chisholm, Andrew N. Gray

**Affiliations:** ^a^Clearwater Forest Analytics, Rapid City, SD 57702; ^b^United States Department of Agriculture, Forest Service, Pacific Northwest Research Station, Corvallis, OR 97331

**Keywords:** forest ecology, large diameter trees, old growth, forestry

## Abstract

Large-diameter trees provide vital ecological functions in forested ecosystems. Old, large-diameter trees may also be vulnerable to climate-driven mortality events, but past work on large tree populations has been geographically limited. Here, we characterize the population of large-diameter trees from two size categories, 50 to 100 cm diameter at breast height (DBH) (medium) and >100 cm DBH (big), within the United States using Forest Inventory and Analysis data. Although populations of big trees are concentrated along the west coast, populations of medium trees are more evenly distributed across the nation. In the western United States, trees >50 cm DBH comprise ~75% of the total carbon stored in live trees, while in the eastern United States they comprise ~20%. Plot remeasurement data indicate that populations of big trees are increasing at an annual rate of 0.49% in the west and 2.9% in the east, and populations of medium trees are increasing at an annual rate of 0.5% in the west and 2.4% in the east. One exception is the Sierra Nevada region, where big trees are declining. Additionally, we observed declines for several individual species. While the overall population trend for large-diameter trees is positive, declines in these species could have localized impacts for the environments in which they occur.

Large-diameter trees are important components of forested ecosystems (1). Research has documented recent die-offs of large-diameter trees (2–4), and mortality rates are projected to rise due to climate change (5). Evidence suggests large-diameter tree mortality may be exceeding recruitment (6, 7), though other studies indicate population stability (8). Because they store substantial amounts of carbon (9, 10), a decrease in large-diameter tree populations could have serious implications for forest carbon storage.

Within the United States, past work on large-diameter tree populations has generally been narrow in scope. Forested land in the United States consists of a matrix of different ownerships and varying levels of legal protection, and few studies have examined demographic trends outside of protected public land. Additionally, few studies documenting mortality measure recruitment (though see refs. 6 and 8) or removals by humans. These limitations make it difficult to determine how large-diameter tree populations (i.e., the number of individuals) may be changing at a national scale.

To conserve large-diameter trees, it is important to identify where they exist, the current rate of population change, and which species may be most vulnerable. The US Forest Service’s Forest Inventory and Analysis (FIA) program, which maintains a national network of over 140,000 repeatedly measured forest research plots spanning all ownerships, is an excellent resource for addressing these questions. FIA is currently nearing completion of a full remeasurement cycle of all plots within the system, enabling accurate estimation of population change with a previously unachievable level of precision. This study leverages recently collected FIA data to characterize populations of trees >50 cm diameter at breast height (DBH) in the United States and determine the current rate and direction of population change.

## Results/Discussion

**Fig. 1.**
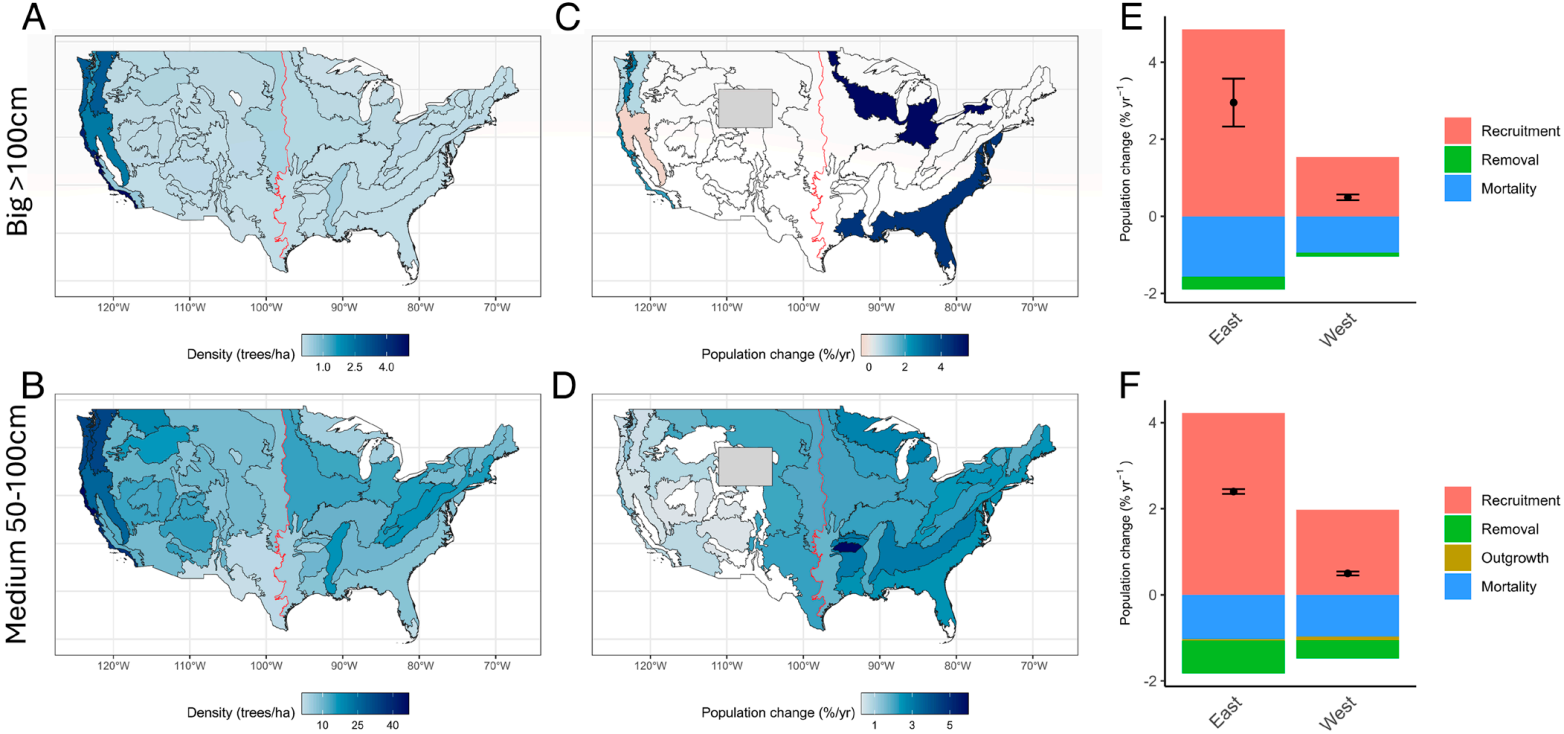
Maps showing current density (*A* and *B*) and change (*C* and *D*) in large-diameter trees on forestland across the United States. Provinces not experiencing statistically significant change are shown in white. Regional delineation is denoted by the red line. Change data unavailable for Wyoming. Panels *E* and *F* show recruitment, mortality, and removal rates for big (*E*) and medium (*F*) trees aggregated by region. Estimates for total population change are denoted by the centered dot ± SE.

The total population of trees >100 cm DBH (“big”) on forestland was estimated to be 81 ± 3.0 million in the western United States (0.82 ± 0.019 trees/ha), compared to 12 ± 1.3 million in the east (0.073 ± 0.0053 trees/ha). Nationally, big tree density was highest along the California coast, though significant populations also existed within the Cascade and Sierra Nevada Ranges (Fig. 1*A*). *Pseudotsuga menziesii* was the most common big tree in the western United States, accounting for 46% of all big trees, while *Quercus rubra* (9%) was the most prevalent big tree in the east. *P. menziesii* (26%) and *Liriodendron tulipifera* (8%) were the most represented 50 to 100 cm DBH (“medium”) trees in the western and eastern United States, respectively. The total population of medium trees was estimated to be 1.5 ± 0.02 billion in the west (16 ± 0.14 trees/ha) and 1.6 ± 0.02 billion in the east (9.9 ± 0.079 trees/ha, Fig. 1*B*).

Large-diameter trees accounted for a substantial portion of the overall carbon stored in live trees. In the west, big trees stored 471 Tg of carbon, 26% of the total live tree carbon pool from that region. Medium trees stored 886 Tg, or 49%. Only 0.1% of all western trees were larger than 100 cm, and 1.9% were between 50 and 100 cm, with the implication that the top ~2% largest trees accounted for 75% of the live tree carbon storage in the western United States. This result suggests the importance of maintaining large-diameter trees in the western United States in order to mitigate climate change. Big and medium trees in the east accounted for a lesser proportion of the total live tree carbon pool, 9.7 Tg (0.5%) and 350 Tg (19%), respectively.

Across the country, populations of large trees were generally stable to increasing. Populations of big and medium trees increased by 0.49% and 0.50% per year, respectively, in the western United States, and 2.9% and 2.4% per year in the eastern United States. At the ecological province level, we observed a statistically significant (*P* < 0.05) increase in the population of big trees in four provinces concentrated along the west coast and two in the eastern United States (Fig. 1*C*). The population of medium trees was significantly increasing in 27 provinces across the country, and there was no significant change in the other nine provinces (Fig. 1*D*). Despite these observed increases, overall current densities were lower than large-tree densities observed in many old-growth forests (11, 12), indicating that the landscape remains a matrix of stands from different successional stages. Further, at observed rates of increase, it would take considerable time for large-tree densities to approach precolonial levels.

Raw counts of sampled big trees used in the change analysis for the east rose from 265 to 316, and 39,111 to 46,267 for medium trees. In the west, sampled counts rose from 17,636 to 18,438 for big trees and 105,216 to 110,494 for medium trees. This increase is proportional to our annualized population change estimates, since most eastern provinces use a 5 to 7 y remeasurement cycle and most western provinces use a 10 y remeasurement cycle.

A significant decline of −0.42% big trees/year was observed in one province, the Sierra Nevada (Fig. 1*C*). This may be due to drought, fire, and bark beetle disturbances in this region 2015 to 2020. To the extent that mortality in some areas has increased in recent years, the FIA averages across multiple remeasurement panels will not fully reflect those recent trends.

**Fig. 2.**
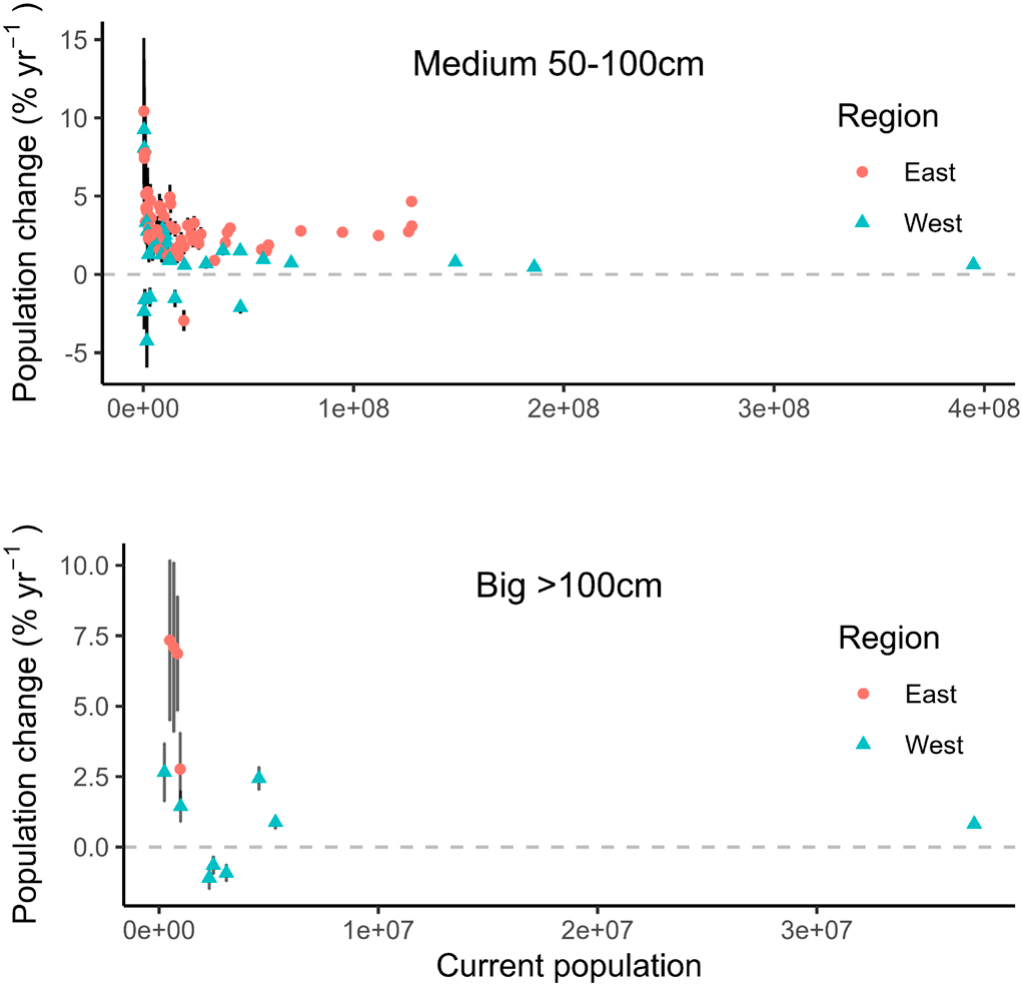
Scatterplot depicting the current population (number of individuals) and population change (annual percent change) for tree species experiencing statistically significant population change. Each point represents an individual species. Species that did not exhibit statistically significant population change are not shown. Error bars depict the SE of the population change estimate. Statistically significant increases in the big size class were observed in nine species and decreases in three species. Increases were observed in the medium size class in 82 species and decreases in seven species. See Dataset S1 for a comprehensive summary of population dynamics by species.

While the overall trend points to increases in large-diameter tree populations, mortality is outpacing recruitment for several species (Fig. 2). Within the big size class, *Pinus lambertiana*, *Abies magnifica*, and *Calocedrus decurrens* are exhibiting statistically significant declines. These species are most abundant in the Sierra Nevada. Statistically significant increases in the big tree population were observed for *P. menziesii, Populus balsamifera, Sequoia sempervirens, Thuja plicata*, and *Picea sitchensis* in the west, and *L. tulipifera*, *Acer saccharinum*, *Taxodium distichum*, and *Quercus virginiana* in the east.

Within the medium size class, statistically significant reductions were observed for seven species nationally. Populations of medium-sized *Abies lasiocarpa*, *Picea engelmannii*, and *Pinus albicaulis*, three species found in western subalpine environments impacted by a complex of native and introduced pests/diseases, were declining. Medium trees from three species located in low-elevation western woodlands—*Quercus douglasii*, *Alnus rhombifolia*, and *Quercus wislizeni*—were also declining, as was *Fraxinus americana*, an eastern species recently affected by an exotic woodborer. In contrast, statistically significant population increases within the medium size class were observed for 21 species in the west and 61 species in the east (Fig. 2 and Dataset S1).

Declines in large-diameter subalpine trees could have long-term impacts, because environmental limitations on productivity in subalpine habitats suggest large trees are unlikely to be quickly replaced after they die. *P. albicaulis,* in particular, exhibited the highest rate of decline. At the current rate, the population of *P. albicaulis* 50 to 100 cm DBH will be halved every 16 y.

The reasons for the proliferation of large-diameter trees in the United States are unclear, but past management may have played a role in creating a demographic bubble of sub-mature trees that are now growing into larger diameter classes. Much of the productive timberland in the western United States was harvested in the previous century, but timber harvests on federal land have slowed during the past three decades (13). In the eastern United States, many forests are maturing after recolonization of former agricultural areas (14). If it is true that the current proliferation of large-diameter trees is due to a demographic bubble created by past human activities, then we would expect the nationwide population increase of large-diameter trees to be driven by elevated recruitment, not by diminished mortality.

An examination of mortality rates indicates that diminished mortality is unlikely to be the cause of the population increase, as our observed mortality rates (Fig. 1 *E* and *F*) for large-diameter trees are roughly equal to or greater than background mortality rates observed in old growth (6, 8). Recruitment rates, on the other hand, range up to 4% (Fig. 1 *E* and *F*), exceeding past observations from unmanaged old-growth forests (6, 8). Rates of removal (i.e., harvest by humans) and outgrowth (trees that have grown out of the medium size class) were generally negligible components of change. Consequently, the relatively high observed mortality rate is being compensated by an even higher recruitment rate. This supports the hypothesis of a demographic bubble created by past human activity.

In general, recent disturbances, such as drought, fire, insects, and disease, have not killed enough large-diameter trees to offset the recruitment of new individuals into these size classes (though our study does not adequately describe the very largest trees on the continent, which grow much larger than 100 cm DBH). However, outliers exist, including the Sierra Nevada region and several subalpine tree species. If this population increase is fueled by a human-created demographic bubble, unmanaged old-growth forests would be unlikely to see the same elevated recruitment and associated population increase. Overall, the increase in large-diameter tree populations across the United States is positive, but region- and species-specific declines may be causes for concern.

## Materials and Methods

To determine the current status of large-diameter tree populations in the United States, we queried FIA data from the most recent publicly available measurement cycle for each state. Within each state, each cycle (i.e., all the plots in a state) takes 5 to 10 y to measure. Due to differences in measurement intensity and reporting time, the completion date of the last cycle varies by state, but ranges from 2019 to 2023. Annualized population change data were obtained for every lower-48 state except Wyoming, which has not undergone a complete remeasurement cycle.

Tree size depends on species and site conditions, so we examined two size classes to ensure the larger trees in a region are captured. Data were summarized by species, ecological province, and region using FIA stratified population estimation (15) to derive estimates and the 95% CI (*SI Appendix*, *Methods*).

## Supplementary Material

Appendix 01 (PDF)

Dataset S01 (XLSX)

Dataset S02 (XLSX)

Dataset S03 (XLSX)

## Data Availability

.csv data have been deposited in FIA Datamart (https://research.fs.usda.gov/products/dataandtools/tools/fia-datamart) (16).
