# Supplementary material for: Populations of large-diameter trees are increasing across the United States
Source: Proc Natl Acad Sci U S A. 2025 Mar 10;122(11):e2421780122. doi: 10.1073/pnas.2421780122 (PMC11929446; doi:10.1073/pnas.2421780122)
Supplement: Supplementary file 1 — Appendix 01 (PDF) [file pnas.2421780122.sapp.pdf]

PNAS

Supporting Information for

Populations of large-diameter trees are increasing across the United States

Paul J Chisholm[1*], Andrew N Gray[2]
1 Clearwater Forest Analytics, Rapid City South Dakota 2 USDA Forest Service PNW Research Station, Corvallis OR
*corresponding author
Email: paul@clearwaterforestanalytics.com

This PDF file includes:

Supporting text
SI References

Other supporting materials for this manuscript include the following:

Dataset S1
Dataset S2
Dataset S3

Supporting Text

Geographic scope

This study included the contiguous lower-48 states of the United States; data from Alaska are incomplete, covering only coastal areas outside designated wilderness, and remeasurement data for Hawaii were not yet available when we conducted the study. Estimates of the current population included all 48 states. Puerto Rico was not included given the small sample size and completely different complement of species from the mainland. However, because a full remeasurement panel had not been completed in Wyoming, we were unable to estimate population change for Wyoming. Forest Inventory and Analysis (FIA) data and documentation were obtained from the national website (https://research.fs.usda.gov/products/dataandtools/tools/fia-datamart). All estimates are based on plots that include forestland, defined as areas of at least 0.4 ha and ≥48 m in width that currently or recently supported at least 10% cover of tree species and are not under nonforest landuses like mowing, pasture, or intensive grazing.

In order to understand geographic differences in large tree dynamics, we summarized the data at the ecoregion level (Cleland et al. 2007). Delineation of ecoregions between east and west roughly followed the 100th meridian; “east” was defined as provinces 255 (Prairie Parkland—Subtropical) and 251 (Prairie Parkland—Temperate) and all provinces east, while “west” included provinces 315 (Southwest Plateau and Plains Dry Steppe and Shrub) and 332 (Great Plains Steppe) and all provinces west.

Temporal scope

For estimates of the current population, such as tree density and total tree population, we used the most recent complete sampling panel available for each state. For this evaluation, 5,479 plots had at least one big tree, and 48,858 plots had a medium tree. Data availability for different states varied due to differences in remeasurement periods and reporting time between FIA units, and more recent data were available for some states than others. All estimates used the most recent data available for each state as of July 1, 2024, which ranged from 2019-2023 depending on the state.

Estimates of change are slightly more constrained, because measurements are necessary at both timepoints, whereas estimates of the current population only require one measurement. Owners denying permission to access their property or safety concerns at the plot location sometimes prevent measurements, so the sample size for change estimates is slightly smaller than for current population estimates. For the change estimation, 4,849 plots contained a big tree, while 45,282 plots contained a medium tree.

Analysis software

Analyses were conducted using R version 4.3.2. Estimates were obtained using the customPSE() function in the rFIA() package (Stanke et al. 2020). A subset of estimates were checked against the USFS EVALIDator (https://apps.fs.usda.gov/fiadb-api/evalidator) tool to ensure consistency.

FIA Sample Design and Population Estimation

Details of the probability-based FIA sample of forested lands, data collection, and equations to generate population-level estimates and variances are found in Bechtold & Patterson (2005). The inventory is based on a randomized systematic grid that spans all U.S. States and affiliated territories. The weight, or contribution of each plot to the population total, is contained in “expansion factors” that account for design differences in plot density as well as the size of post-classified strata each plot occurs in. Post-stratification is intended to increase the precision of estimates and has been used for decades by FIA and forest inventories around the world (Frayer and Furnival 1999, Tomppo et al. 2010). Post-stratification has a strong theoretical basis (Cochran 1963, Särndal et al. 1992), and is the topic of extensive applied research (Katila and Tomppo 2002, Westfall et al. 2021). Post-stratification is also employed to create homogeneous response groups for sub-populations of sample plots that have similar rates of nonresponse (e.g.,

access denied or inaccessible forestland) (Patterson et al. 2012). While a wide range of stratification schemes are possible, post-stratification does not bias the values being estimated, especially if the samples are distributed proportionally as in the FIA inventory (Brigazzi et al. 2018, McConville et al. 2020). Comparisons of different stratification approaches shows that estimates are always within the 95% confidence intervals of each other (e.g., Table 1 in Westfall et al. 2019).

Within each state in the FIA inventory, plots are assigned to independent strata expected to reduce within-strata variance in plot estimates (e.g., area of forestland), using spatial layers of classified remote sensing, elevation, climate, and/or ownership. Plot weights are simply the number of sampled forest and fully nonforest plots divided by the area of each stratum. Strata and estimation “evaluations” are defined for a complete measurement cycle of multiple annual panels within a state, with new evaluations created as each new annual panel of plots is measured (or remeasured). The evaluation tables to estimate population totals, means, and variances are identified with the prefix “POP” in the online FIA database, with the strata definitions in the POP_STRATUM table (https://apps.fs.usda.gov/fia/datamart/CSV/ENTIRE_PLOT_STRATUM.csv). Identification numbers for the specific evaluations used in our change analysis (the EVALID) can be found in supporting Dataset S3.

Trees are sampled with different areas within FIA plots, so every tree is assigned a per-hectare weight based on the area sampled, and that weight is used for all estimates of numbers and densities of trees. Annualized estimates (i.e., trees per year) are calculated using the remeasurement period for each plot, which ranges from 5 to 10 years depending on the FIA unit. Estimates of total number of trees meeting species or size criteria combined the tree-level sample weights and the stratification weights using equation 4.13 in Bechtold and Patterson (2005) which applies calculations from equations 4.8, 4.9, and 4.11. Variance was estimated with equation 4.14; the square root of the variance defines the standard error of the mean or total, and the 95% confidence interval is obtained by multiplying the standard error by ±1.96, which accounts for 95% of the standard normal distribution (i.e., the Z distribution) used for large random samples (Bechtold and Patterson 2005, Westfall et al. 2022). Similarly, the estimate divided by the standard error yields a z-score from which the likely p-value can be obtained. We tested for statistical significance by generating 95% confidence intervals, i.e., a significance threshold of $\alpha = 0.05$. Estimates of changes in numbers of trees or density were determined to be significant if the 95% confidence interval around the estimate did not include 0. These estimates of totals and standard errors were obtained for desired species, species groups, and size criteria for desired domains of interest, including ecoregion provinces and national regions (east or west).

Estimates of tree density (trees per ha) were obtained using ratio of means by dividing the estimated total number of trees by the estimated area of forest land in a unit of interest, and the variance of the ratio that accounts for the covariance between the estimates (equations 4.16, 4.18, and 4.19 in Bechtold and Patterson 2005).

Estimates of growth, mortality, and removals were based on remeasured and reconciled trees in the GRM tables in the FIA database. Mortality trees were defined as those that met the size threshold and were alive at time 1, and were dead at time 2. Recruitment trees were defined as those that did not meet the size threshold at time 1, but met the size threshold and were alive at time 2. Removal trees were defined as those that were alive and met the size threshold at time 1, and were harvested by time 2. Outgrowth is only applicable to the medium size class. Outgrowth trees were those that were alive and between 50-100cm DBH at time 1, and alive but >100cm DBH at time 2. Annual rates of mortality, recruitment, removal, and outgrowth (%/year) were obtained by taking the estimated annual rate (i.e., trees/year from the measurement interval on each plot) and dividing by the current total population estimate.

Dataset S1 (separate file). Data showing the sample size (number of trees and number of plots) used to generate population-level estimates, as well as estimates and associated confidence intervals for each species

Dataset S2 (separate file). Data showing the raw sampled tree counts at time 1 and time 2.

Dataset S3 (separate file). Data showing the evaluation information for the specific evaluations used in our change analysis. This information can be used to look up which plots were used in our change analysis, and the post-stratification methods used to generate population-level estimates.

SI References

1. Birigazzi, L., J.G.P. Gamarra, and T.G. Gregoire. 2018. Unbiased emission factor estimators for large-area forest inventories: domain assessment techniques. Environmental and Ecological Statistics 25:199-219.
2. Cleland, D.T., Freeouf, J.A., Keys, J.E., Nowacki, G.J., Carpenter, C.A., and McNab, W.H. 2007. Ecological Subregions: Sections and Subsections for the conterminous United States. Gen. Tech. Report WO-76D (A.M. Sloan, cartographer). Washington, DC: U.S. Department of Agriculture, Forest Service.
3. Cochran, W.G. 1963. Sampling Techniques, 2nd edition. John Wiley & Sons, New York.
4. Frayer, W.E. and G.M. Furnival. 1999. Forest survey sampling designs: a history. Journal of Forestry 97:4-10.
5. Katila, M. and E. Tomppo. 2002. Stratification by ancillary data in multisource forest inventories employing k-nearest neighbour estimation. Canadian Journal of Forest Research 32:1548-1561.
6. McConville, K.S., G.G. Moisen, and T.S. Frescino. 2020. A Tutorial on Model-Assisted Estimation with Application to Forest Inventory. Forests 11:244.
7. Patterson, P.L., J.W. Coulston, F.A. Roesch, J.A. Westfall, and A.D. Hill. 2012. A primer for nonresponse in the US forest inventory and analysis program. Environmental Monitoring and Assessment 184:1423-1433.
8. Särndal, C.E., B. Swensson, and J.H. Wretman. 1992. Model assisted survey sampling. Springer-Verlag, New York.
9. Stanke, H., Finley, A.O., Weed, A.S., Walters, B.F. and Domke, G.M., 2020. rFIA: An R package for estimation of forest attributes with the US Forest Inventory and Analysis database. *Environmental Modelling & Software*, *127*, p.104664.
10. Tomppo, E., T. Gschwantner, M. Lawrence, and R.E. McRoberts, eds. 2010. National forest inventories: pathways for common reporting. Dordrecht: Springer. 612 p.
11. Westfall, J.A., J.W. Coulston, G.G. Moisen, and H.-E. Andersen. 2022. Sampling and Estimation Documentation for the Enhanced Forest Inventory and Analysis Program. U.S. Department of Agriculture, Forest Service, Northern Research Station, Madison, WI. GTR-NRS-207. https://research.fs.usda.gov/treesearch/65551
12. Westfall, J.A., A.J. Lister, C.T. Scott, and T.A. Weber. 2019. Double sampling for post-stratification in forest inventory. European Journal of Forest Research 138:375-382.
13. Westfall, J.A., A.J. Lister, J.W. Coulston, and R.E. McRoberts. 2021. Realized and potential efficiency for post-stratified estimation in a national forest inventory. Canadian Journal of Forest Research 51:1450-1457
